# Paraneoplastic Mononeuritis Multiplex: A Unique Presentation of Non-Hodgkin Lymphoma

**DOI:** 10.7759/cureus.2885

**Published:** 2018-06-26

**Authors:** Abdul Ahad E Sheikh, Abu Baker Sheikh, Usman Tariq, Fasih Sami Siddiqui, Waseem T Malik, Haris M Rajput, Imran Ahmad

**Affiliations:** 1 Student, Shifa College Of Medicine, Islamabad, PAK; 2 Internal Medicine, University of New Mexico, Albuquerque, USA; 3 Research Assistant, Yale University School of Medicine, New Haven, USA; 4 Neurology, Shifa International Hospital, Islamabad, PAK; 5 Neurology, Pakistan, Islamabad, PAK; 6 Pathology, Shifa International Hospital, Islamabad, PAK

**Keywords:** mononeuritis multiplex, paraneoplastic syndrome, non-hodgkin lymphoma

## Abstract

Mononeuritis multiplex (MM) is a common variant of a peripheral neuropathy which is characterized by neurological discrepancies that afflict two noncontiguous nerve systems. It is mostly associated with systemic illnesses such as diabetes mellitus, vasculitis, systemic lupus erythematosus (SLE), viral infections including human immunodeficiency virus (HIV) and paraneoplastic syndromes. Lymphoma is a common antecedent to paraneoplastic syndromes that cause peripheral neuropathies but a specific presentation of MM is a rare predicament per our literature analysis.

## Introduction

Peripheral neuropathies encompass a wide array of clinical presentations, in virtue of a diverse lineup of diseases that afflict the peripheral nervous system. Mononeuritis multiplex (MM) differs from other neuropathies due to its deviation from their normal clinical presentation [1]. It is characterized by a painful and disproportional neuropathy which embroils the sensory and motor nervous systems. It is clinically distinguished due to its pattern of afflicting two separate nerve areas simultaneously. MM worsens with time, owing to a worsening of the precipitating disease process. This most commonly includes systemic disorders such as diabetes mellitus, vasculitis, amyloidosis, systemic lupus erythematosus (SLE), viral infections such as acquired immunodeficiency virus (AIDS), hepatitis, parvovirus B19, multiple compression neuropathies and paraneoplastic syndromes [2-3]. The most prevalent paraneoplastic syndromes in lymphoma are cerebellar degeneration and limbic encephalitis [4]. We report the case of a patient with non-Hodgkin lymphoma (NHL) who presented to our clinical setting with the paraneoplastic manifestation of MM, which is a rare finding in the setting of lymphoma per our literature review.

## Case presentation

A 25-year-old female with gestational amenorrhea for 32 weeks presented to our outpatient department with complaints of a recurring headache along with pain and weakness in the legs for the past seven months. The headache was described as a bilateral, dull and persistent pain that fluctuated between mild to moderate in intensity. She also described neck stiffness along with her chief complaints but denied any nausea, vomiting, and changes in gait or memory. The pain in her legs waxed and waned over time, although progressively increasing in intensity with each passing episode. At the outset of this predicament, pain was localized to her left leg, eventually became symmetrical and later progressed to afflict both arms. She denied numbness or paresthesia. She was eventually brought to our clinical setting following an aggravation of her symptoms over the previous two weeks that lead to a restriction in mobility. At the time of this presentation, she also complained of double vision that was gradually worsening. She also added that she experienced fluctuating fevers, undocumented weight loss, and episodes of night sweats for the last four months. 

Initial assessment found the patient to be alert and well-oriented, with a Glasgow Coma Scale score (GCS) of 15/15, albeit thin, emaciated, and noticeably distressed due to her clinical predicament. Her heart rate (HR) was 102/minute with a respiratory rate (RR) of 18/minute, a temperature of 98.4°F and a blood pressure (BP) of 110/175 mm Hg. A neurological examination revealed generalized weakness and a bilaterally diminished muscle tone. A strength assessment revealed that she had reduced power in her upper (right arm; 2/5, left arm; 4/5) and lower (right leg; 1/5, left leg; 3/5) extremities. There was a complete absence of all deep tendon reflexes except the biceps. A comprehensive ophthalmological exam demonstrated normal visual acuity, with notable issues in the right eye which included ptosis, fixed and dilated pupil, and diplopia which manifested with the right-sided gaze. A funduscopic examination showed normal definitions. She also had a bilateral facial nerve palsy which affected the lower half of the face, along with reduced sensation along the distribution of the maxillary and mandibular divisions of the trigeminal nerve. This deficit was more pronounced on the right side of the face. On abdominal examination, an appendectomy scar was visible on the right iliac fossa. The abdomen was protuberant, soft and non-tender. Bowel sounds were audible and inguinal lymph nodes were not palpable. 

Initial laboratory investigations were within normal limits, with the exception of an elevated erythrocyte sedimentation rate (ESR) of 128 millimeters/hour. A magnetic resonance imaging (MRI) scan of the brain ruled out any local pathology that could explain her neurological deficits. A cerebrospinal fluid (CSF) analysis following a lumbar puncture revealed a white blood cell (WBC) count of 1500 with neutrophilic predominance, which leads to the initiation of empirical therapy for bacterial meningitis. Two days after the initiation of antibiotic therapy, she complained of dull abdominal pain and fullness. She was investigated with an abdominal ultrasound which revealed a thickened descending colon with a well-defined heterogeneous lesion measuring 89 mm by 94 mm in the left adnexal region that impinged on the uterus and urinary bladder due to its mass effect. The lesion was further investigated using a computed tomography (CT) scan which disclosed a circumferential mural thickening in the distal third of the descending colon with no luminal narrowing at the site. Multiple enlarged para-aortic lymph nodes were also appreciated, with the largest measuring 17 mm by 10 mm at the level of the left renal hilum.

**Figure 1:**
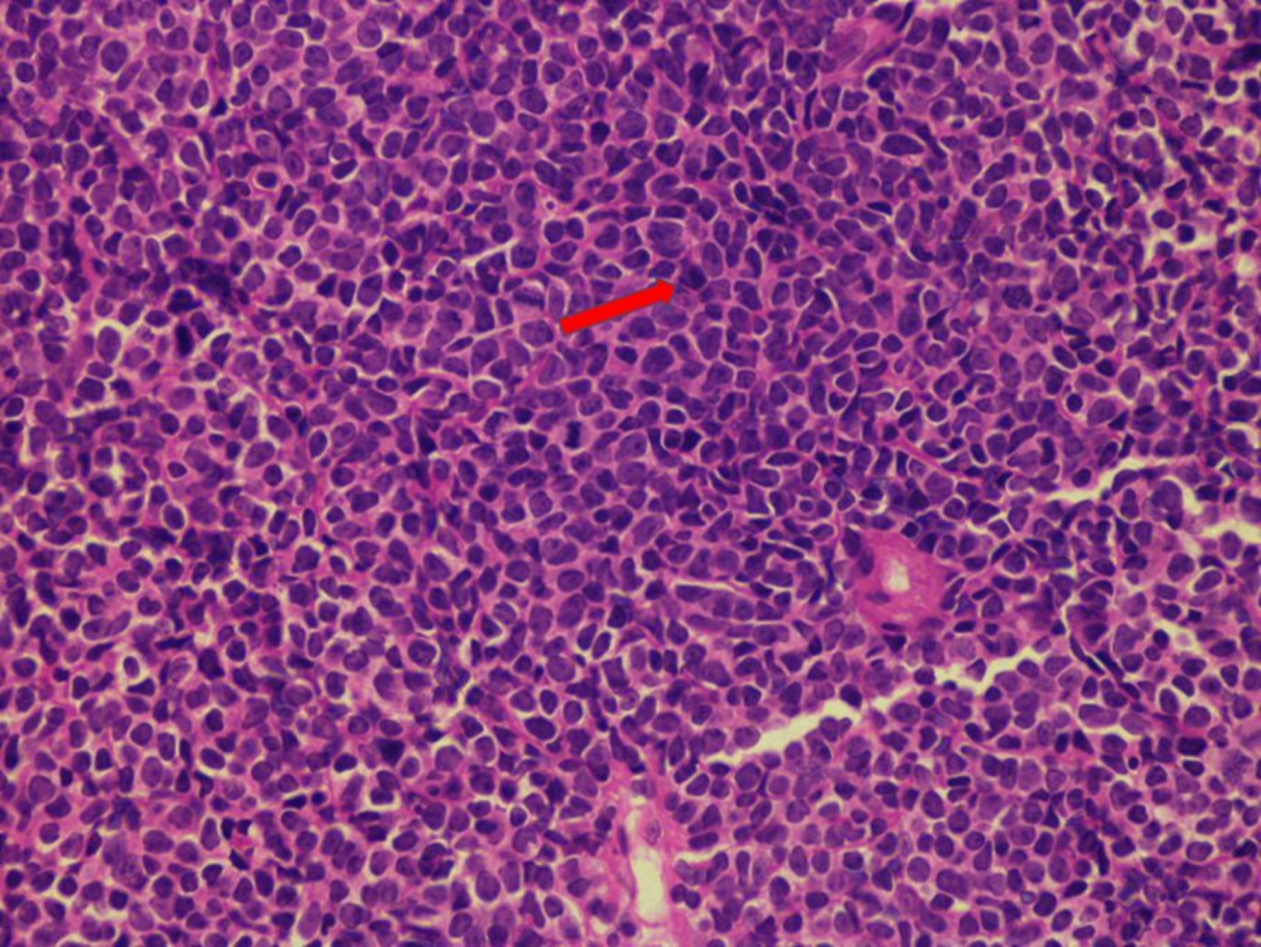
Histological segment of a lymph node illustrated via a hematoxylin and eosin (H&E) stain The H&E stain depicting nodal invasion by immature cells that are characterized by scarce cytoplasmic distribution, well-defined nuclei, and prominent chromatin. Such findings allude to the presence of a lymphoblastic lymphoma.

**Figure 2:**
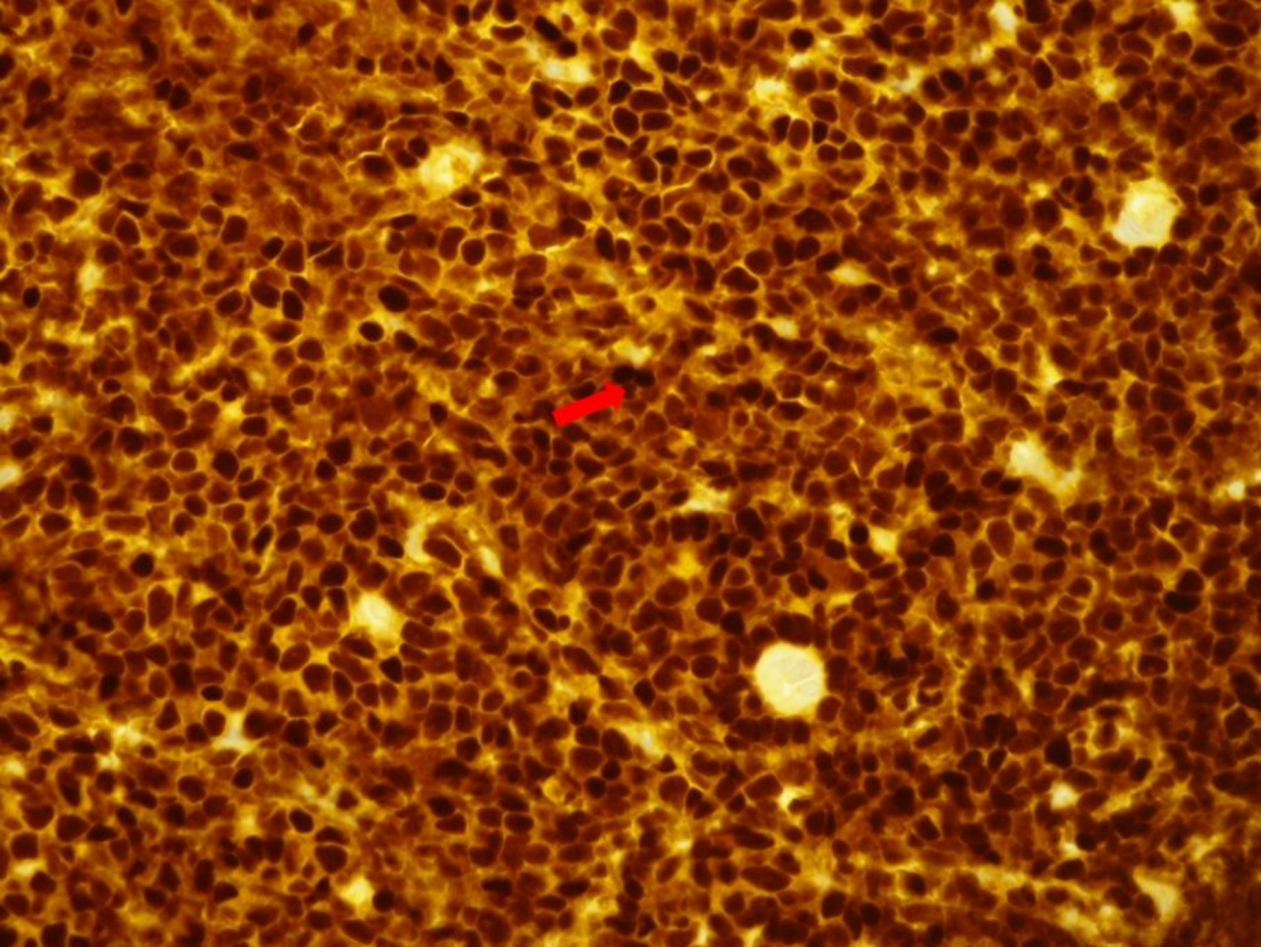
Immunohistochemical stain of the lymph node depicting tumor cells that are positive for anti-TdT antibodies

**Figure 3:**
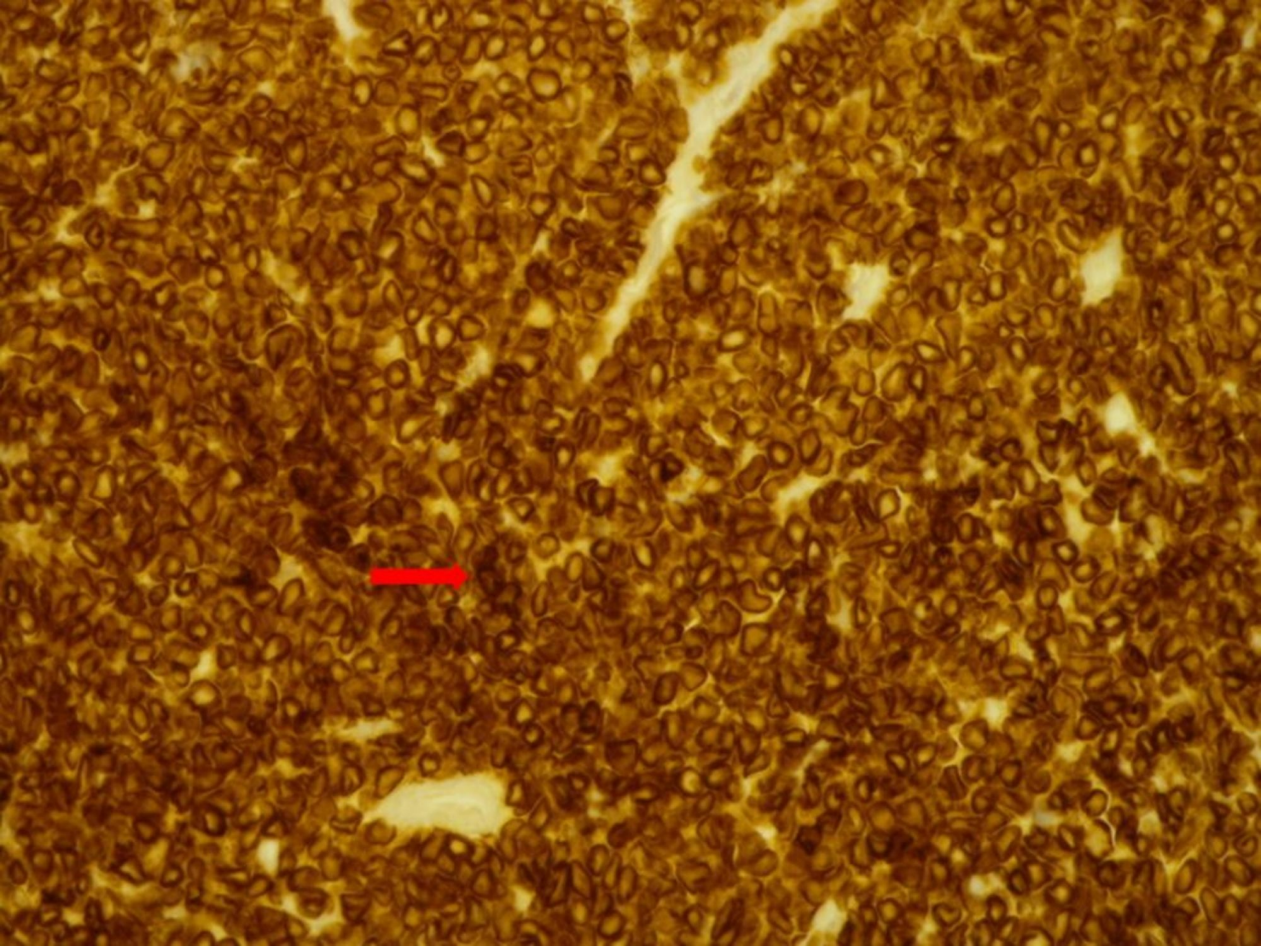
Immunohistochemical stain of the lymph node depicting tumor cells that are positive for anti-CD3 antibodies

Following patient stabilization, the adnexal mass was biopsied which showed an atypical infiltrate composed of small to medium cells exhibiting immature chromatin, irregular nuclear folds, and increased mitosis. Immunohistochemistry showed this infiltrate to be CD3 (+), TdT (+), CD99 (+), PAX-5 (-) and CD20 (-), consistent with precursor T cell lymphoblastic lymphoma (Figures 1-3).

In lieu of her newly diagnosed malignancy, we suspected that her neurological deficits could be attributed to a paraneoplastic syndrome. A workup for autoimmune etiologies was unrevealing based on a negative serum electrophoresis, negative anti-ganglioside profile and a negative anti-neuronal profile (Tables 1-3).

**Table 1:** Autoimmune profile ANA: anti-nuclear antibody; Anti-dsDNA: anti-double stranded deoxyribonucleic acid antibody; ANCA: anti-neutrophil cytoplasmic antibody; ENA: extractable nuclear antigen antibodies.

Antibody	Result
ANA	Negative
Anti-dsDNA	Negative
ANCA	Negative
ENA Panel	Negative

**Table 2:** Anti-neuronal profile CRMP: collapsin response-mediator protein; PNMa2: paraneoplastic Ma2; ANNA: anti-neuronal nuclear autoantibody; PCA: Purkinje cell cytoplasmic antibody; AGNA: anti-glial nuclear antibody.

Antibody	Result
Anti-Amphiphysin Antibody	Negative
Anti-CRMP Antibody	Negative
Anti-PNMa2 Antibody	Negative
Anti-ANNA 2 Antibody	Negative
Anti-PCA 1 Antibody	Negative
Anti-ANNA 1 Antibody	Negative
Anti-Recoverin Antibody	Negative
Anti-AGNA 1 Antibody	Negative
Anti-Titin Antibody	Negative

**Table 3:** Anti-ganglioside profile GM: ganglioside monosialic; GDA: guanine deaminase; GTB: glycosyltransferase B; GQ: ganglioside quad; NMDA: N-methyl-D-aspartate.

Antibody	Result
Anti-GM1 Antibody	Negative
Anti-GM2 Antibody	Negative
Anti-GM3 Antibody	Negative
Anti-GDA Antibody	Negative
Anti-GTB Antibody	Negative
Anti-GQ1b Antibody	Negative
Anti-NMDA Antibody	Negative

The patient eventually underwent a nerve conduction study that revealed severe sensory-motor axonal neuropathy involving the left tibial, left peroneal and right facial nerve.

An eventual diagnosis of paraneoplastic MM was formulated based on a combination of the physical findings and documented nerve damage that simultaneously affected two separate nerve areas. She was treated with plasmapheresis which eventually led to a resolution of her neurological discrepancies. The patient remains asymptomatic till date from a neurological standpoint.

## Discussion

Paraneoplastic neurological syndromes (PNS) can be explained as distant ramifications of a malignancy that cannot be attributed to the local annexation of a tissue by either the primary pathology or its resultant metastasis. The presence of neurological deficits in the absence of factors such as infection or impaired blood supply to the local tissue, incongruences in metabolic processes, systemic toxicity, ectopic hormone production, or tumor stimulated blood disorders should elude a physician to the presence of a PNS. These deficits are rare in the setting of an underlying malignancy and although the link with a neoplastic growth is well known, only 1% of patients with cancer will eventually develop this clinical entity [5-6].

The clinical presentation of a PNS depends on the kind of malignancy and the resultant damage to the nerve. Regardless of the variant, a PNS could occur due to an injury to either the central nervous system or the peripheral nervous system. The severity of the symptoms may not correlate with the scale of the assault on a nerve tissue. This can be illustrated from the fact that some cases of PNS could present with an expeditious course, which renders the patient incapacitated in terms of motor and/or sensory functionality, within a period of weeks. However, other forms could present with a gradually waxing and waning course such as in our patient [5].

The cornerstone in the theory explaining the pathophysiology of a PNS is the synthesis and subsequent expression of a neuronal protein, referred to as an onconeural antigen, upon a tumor cell. The onconeural antigen is also expressed as a normal protein by the cellular structures of normal nerve tissues. Despite this notion, the intact immune system falsely acknowledges the antigen as foreign and mounts a response against it. Dendritic cells phagocytize the tumor cells that carry this antigen, which may or may not be disease-specific, and presents it to the CD4+, CD8+ T cells and B lymphocytes. The activated CD8+ T cells and the B lymphocytes (through the formation of antibodies) may halt the advance of the tumor itself. However, the nervous system often becomes the site of collateral damage, owing to the expression of the antigen in the healthy nerve tissues. The CD8+ T cells and B lymphocytes may intrude the blood-brain barrier which may explain the symptoms that manifest following an upper motor neuron insult [6]. However, less than 50% of the patients with a PNS are positive for an anti-onconeural antibody [7]. A similar finding was also appreciated in our patient. 

MM is an asymmetrical, asynchronous and painful form of a PNS which usually affects two or more nonadjacent motor and/or sensory nerves [8]. The most prevalent paraneoplastic syndromes in lymphoma are cerebellar degeneration and limbic encephalitis [5]. In our literature review, we found a scarcity in the number of case reports that describe a presentation similar to MM as the initial clinical demonstration of an NHL, which underlines the rarity of this clinical picture [9-11]

Vallat et al. described 13 cases of NHL associated with peripheral neuropathies. The basis of the resultant neurological deficiencies was described by placing the study pool into four different groups. Group I included patients where peripheral neuropathy was explained by a direct invasion of the neoplastic cells into a peripheral nerve, as supported by the findings on an autopsy, a nerve biopsy or both. Group II included patients where the neurological defect was attributed to a secretant NHL which leads to monoclonal dysglobulinemia. Peripheral neuropathy in group III was attributed to an autoimmune-mediated process while the patients in group IV had neurological discrepancies without an association to prior causes. The findings in these patients were attributed to be a paraneoplastic materialization of the tumor [12].

Our patient had a negative serum electrophoresis which ruled out a monoclonal dysglobulinemia. A negative autoimmune profile ruled out autoimmune processes. This left us with only two possibilities, a direct tumor invasion to the nerves or a paraneoplastic syndrome associated with the tumor. The complete recovery of the patient following plasmapheresis convincingly directs the diagnosis towards a paraneoplastic syndrome and negates tumor invasion as a possible diagnosis.

## Conclusions

NHL and its association with paraneoplastic syndromes are well documented, with the most common variants being cerebellar degeneration and limbic encephalitis. Our patient initially presented with a paraneoplastic syndrome that manifested as MM which is rarely observed in patients with NHL. The rarity of this predicament supplemented with negative autoimmune and anti-neuronal profiles made the case a diagnostic challenge. We reiterate the importance of having a high degree of clinical suspicion for an underlying lymphoma in patients with signs and symptoms of MM, which can lead to an earlier diagnosis and initiation of prompt treatment.
